# *LEP* Gene Promotes Milk Fat Synthesis via the JAK2-STAT3 and mTOR Signaling Pathways in Buffalo Mammary Epithelial Cells

**DOI:** 10.3390/ani14162446

**Published:** 2024-08-22

**Authors:** Ruixia Gao, Qunyao Zhu, Lige Huang, Xinyang Fan, Xiaohong Teng, Yongwang Miao

**Affiliations:** Institute of Animal Genetics and Breeding, College of Animal Science and Technology, Yunnan Agricultural University, Kunming 650201, China; malegreshell@126.com (R.G.); 18213524162@163.com (Q.Z.); 18838969693@163.com (L.H.); xinyangfan1@ynau.edu.cn (X.F.); 13698786782@163.com (X.T.)

**Keywords:** buffalo *LEP* gene, transcript variants, gene function, milk fat synthesis, population genetic analysis

## Abstract

**Simple Summary:**

Milk fat is an important component of milk and dairy products. Previous studies have shown that leptin (LEP) regulates lipid metabolism in cattle, while its role, especially its impact on milk fat synthesis in buffalo mammary epithelial cells (BuMECs), remains unknown. Understanding the leptin gene’s function can provide insights into the genetic basis of lactation traits in buffalo and help enhance their milk production performance. In this study, we isolated and identified two transcript variants of the buffalo *LEP* gene, both of which positively regulate milk fat synthesis. Our results indicate that *LEP* transcript variant 2, in particular, plays a more significant role in regulating milk fat in buffalo compared to variant 1, offering a basis for elucidating the functional role of *LEP* in the buffalo mammary gland.

**Abstract:**

Leptin (LEP), a protein hormone well-known for its role in metabolic regulation, has recently been linked to lipid metabolism in cattle. However, its function in buffalo mammary glands remains unclear. To address this issue, we isolated and identified the *LEP* gene and conducted experiments to investigate its function in buffalo mammary epithelial cells (BuMECs). In this study, two transcript variants of *LEP*, designated as *LEP*_X1 and *LEP*_X2, were identified. The coding sequences (CDS) of *LEP*_X1 and *LEP*_X2 are 504 bp and 579 bp in length, encoding 167 and 192 amino acid residues, respectively. Bioinformatics analysis revealed that LEP_X2 is a hydrophobic protein with an isoelectric point below 7 and contains a signal peptide, while LEP_X1 is hydrophilic and lacks a signal peptide. Our study found that *LEP* gene expression in lactating BuMECs was significantly higher than in non-lactating cells, with *LEP*_X2 expression remarkably higher than *LEP*_X1 in lactating BuMECs. Overexpression of both *LEP*_X1 and *LEP*_X2 significantly promoted the expression of genes related to milk fat synthesis in lactating BuMECs, including *STAT3*, *PI3K*, *mTOR*, *SCD*, and *SREBF1*, accompanied by an increase in cellular triglycerides (TG). Interestingly, *LEP*_X2 overexpression significantly suppressed *LEP*_X1 expression while increasing intracellular TG concentration by 12.10-fold compared to *LEP*_X1 overexpression, suggesting an antagonistic relationship between the two variants and supposing *LEP*_X2 plays a dominant role in milk fat synthesis in lactating BuMECs. Additionally, four nucleotide substitutions were identified in the buffalo *LEP* CDS, including a nonsynonymous substitution c.148C>T (p.Arg50Cys), which was predicted to decrease the stability of the LEP protein without affecting its function. These results collectively underscore the significant role of *LEP* in milk fat synthesis and can provide a basis for molecular breeding strategies of buffalo.

## 1. Introduction

Leptin (LEP) is a pivotal extracellular signaling hormone encoded by the *LEP* gene, originally identified in mice through positional cloning as the *ob* gene [1]. Leptin functions primarily as a critical sensor for energy balance and metabolism by binding to the membrane proximal cytokine receptor homology domain (CRH2) of the leptin receptor (LEPR) [2,3]. It influences energy metabolism, which is supported by blood lipids such as phospholipids, cholesterol, and triglycerides, thereby impacting both endocrine system functions and intracellular signaling pathways [4]. In sheep, leptin is produced during various stages of mammary gland development, suggesting its role as a paracrine factor that influences mammary gland growth, development, and function [5].

Single nucleotide polymorphisms (SNPs) in the *LEP* gene have been shown to significantly affect milk yield and quality traits across various species [6]. In cattle, a nonsynonymous amino acid substitution (A59V) impacts key traits such as age at first calving and milk yield [7,8]. Furthermore, Mahrous et al. [9] highlighted the effects of *LEP* SNPs on mRNA splicing, protein stability, and gene function in Egyptian river buffalo.

In mice, the *LEP* gene is located on chromosome 6, with a coding sequence (CDS) of 504 bp encoding 167 amino acids and including regulatory elements in the promoter region [10,11]. In cattle, the *LEP* gene resides on chromosome 4 and has two transcript variants, with CDS lengths of 504 bp and 501 bp (accession number: NM_173928.2, XM_010804455.4), encoding 167 and 166 amino acid residues, respectively. In buffalo, the *LEP* gene is located on chromosome 8 and comprises three transcript variants (accession number: NM_001290901.1, XM_044946369.2, XM_044946370.2). Table S1 provides a detailed overview of *LEP* gene transcript variants in other Bovidae species. These variants, generated through alternative splicing of primary RNA, result in differences in protein structure, function, and cellular localization.

Buffaloes are categorized into two main types: river buffalo (2n = 50) and swamp buffalo (2n = 48), which differ in chromosome number, biological characteristics, and behavior [12]. River buffaloes are primarily used for dairy production, while swamp buffaloes are employed for labor purposes. This study focuses on two specific buffalo breeds: the Binglangjiang (BLJ) buffalo, a river-type breed from Tengchong City, Yunnan Province, China, known for its high milk yield (average annual yield of 2452.2 ± 553.8 kg per lactation) and elevated milk protein (4.60%) and fat content (6.82%) [13,14,15], and the Dehong buffalo, a swamp buffalo from Dehong Prefecture, Yunnan Province, China, recognized for its gentle temperament and high fiber digestibility, used primarily for labor [16].

The aim of this study is to clone and characterize the *LEP* transcript variants in buffalo mammary gland during lactation and non-lactation periods and to elucidate the role of these transcript variants in milk fat synthesis and their molecular mechanisms. This study is expected to provide profound insight into the role of the *LEP* gene in buffalo milk fat synthesis and contribute to advancements in buffalo dairy production.

## 2. Materials and Methods

### 2.1. Sample Collection

In this study, tissue samples were collected from ten healthy female BLJ buffaloes (river type, 4 years old, the third parity) under consistent management conditions. Five buffaloes were in the lactation period (60 days postpartum), and the other five were in the dry-off period (60 days before parturition). Mammary gland tissue samples were obtained using the puncture technique [17] and preserved in liquid nitrogen, and subsequently stored at −80 °C.

For SNPs detection, blood samples were collected from 144 BLJ buffaloes and 246 Dehong buffaloes (swamp type) with no direct kin relationship. Samples were collected in anticoagulation tubes and stored at −20 °C for subsequent DNA extraction and analysis.

### 2.2. Gene Cloning and Identification

Total RNA was isolated and purified from the mammary gland tissues and mammary epithelial cells of buffaloes using RNAiso Plus (TaKaRa, Dalian, China). The integrity and concentration were assessed by agarose gel electrophoresis and a NanoDrop 2000 UV–Vis spectrophotometer (Thermo Fisher Scientific, Waltham, MA, USA), respectively. cDNA was synthesized using Oligo(dT)_18_ (500 μg/mL) and the M-MLV reverse transcription kit (TaKaRa, Dalian, China), diluted to 100 ng/µL, and stored at −80 °C for further analysis.

Based on the mRNA sequence of buffalo *LEP* (Table S1, accession No. XM_044946369.2, X1; NM_001290901.1, X2), primers for isolating and identifying transcript variants of the *LEP* gene were designed using Primer Premier 5.0 software in Table S2 [18]. The PCR reaction system consisted of 25 µL total volume, including 12.5 µL of 2× Hieff Canace^®^PCR Master Mix (Dye), 1.25 µL of each primer (10 µmol/L), 1.25 µL of 100 ng/µL cDNA template, and 8.75 µL of sterile water. The PCR procedure included an initial pre-denaturation at 95 °C for 3.5 min, followed by 35 cycles of denaturation at 95 °C for 10 s, annealing at 64.7 °C for 30 s, and extension at 72 °C for 30 s, with a final extension at 72 °C for 5 min. The PCR products were detected by 1.5% agarose gel electrophoresis, purified, and cloned into a pMD18-T vector (TaKaRa, Dalian, China). Ten monoclonal clones of each transcript variant were selected and sequenced bidirectionally by Shanghai Sangon Biotech Co., Ltd, Shanghai, China.

### 2.3. Bioinformatics Analysis

The sequences obtained were verified and output using SeqMan and EditSeq (DNASTAR) [19]. The open reading frame (ORF) was identified using ORF Finder to obtain the *LEP* CDS (https://www.ncbi.nlm.nih.gov/orffinder/, last accessed on 10 February 2024). Homology searches were conducted using the BLAST program in the NCBI database (https://blast.ncbi.nlm.nih.gov/Blast.cgi, last accessed on 10 February 2024). We performed an exhaustive comparison of the *LEP* CDS obtained in this study with sequences from the NCBI database to identify the commonalities and differences. The sequence information is detailed in Table S1. Codon usage bias of the *LEP* gene was analyzed using CodonW [20] (http://codonw.sourceforge.net/, last accessed on 10 February 2024). The physicochemical characteristics of the *LEP*-encoded protein were predicted using ProtParam [21] (https://web.expasy.org/protparam/, last accessed on 10 February 2024). Hydrophobicity, signal peptide, and transmembrane regions were predicted using ProtScale [21] (https://web.expasy.org/protscale/, last accessed on 10 February 2024), SignalP-4.1 [22] (https://services.healthtech.dtu.dk/service.php?SignalP-4.1, last accessed on 10 February 2024), and TMHMM-2.0 [23] (https://services.healthtech.dtu.dk/service.php?TMHMM-2.0, last accessed on 10 February 2024), respectively. The conserved domain of the *LEP* gene was analyzed using the CD-search tool in the NCBI database (https://www.ncbi.nlm.nih.gov/cdd/?term=, last accessed on 10 February 2024). Amino acid sequences were submitted to the MEME suite website (https://meme-suite.org/meme/, last accessed on 10 February 2024) to identify motif compositions [24].

The structural analysis of the LEP between buffalo and Bovidae species was studied using the following online tools: secondary structures were predicted with SOPMA [25] (http://npsa-pbil.ibcp.fr/, last accessed on 10 February 2024) and tertiary structures with SWISS-MODEL [26] (http://swissmodel.expasy.org/, last accessed on 10 February 2024). Genome annotation files for the *Bos*, *Ovis*, and *Mus* genera were obtained from the NCBI database (https://www.ncbi.nlm.nih.gov/datasets/, last accessed on 10 February 2024). The structure of the transcriptional regions was visualized using the Gene Structure Display Server [27] (http://gsds.gao-lab.org/, last access: 10 February 2024). Motifs, conserved domains, and phylogenetic tree were visualized using TBtools [28]. The identity of amino acid sequences in each species was analyzed with Megalign [29].

Additionally, the biological processes and functions of the *LEP* gene were predicted using InterProScan [30] (http://www.ebi.ac.uk/interpro/search/sequence/, last accessed on 10 February 2024). Protein interactions involving LEP were predicted using the STRING software [31] (https://cn.string-db.org/, last accessed on 10 February 2024). GO enrichment analysis was conducted using the DAVID online tool [32] (https://david.ncifcrf.gov/, last accessed on 10 February 2024).

### 2.4. Overexpression Vector Construction

To construct overexpression vectors for the *LEP* gene, primers with double restriction sites were designed to amplify the *LEP*_X1 and *LEP*_X2. The amplified genes were then inserted into the pEGFP-N1 vector (CLONTECH Laboratories, Inc, Palo Alto, CA, USA) to create the pEGFP-N1-LEP_X1 and pEGFP-N1-LEP_X2 overexpression vectors. The recombinant vectors were verified by bidirectional sequencing.

### 2.5. Cell Culture and Transduction

To investigate the cellular functions of *LEP*, BuMECs were isolated from the mammary gland tissues of lactating BLJ buffaloes, 60-day postpartum. The isolation and identification of BuMECs were carried out following the procedures previously described by our group [33,34,35]. After purification, the BuMECs were cultured in DMEM/F12 (Gibco, New York, NY, USA) supplemented with 10% FBS (Gibco), 5 μg/mL insulin (Sigma St. Louis, MO, USA), 5 μg/mL hydrocortisone (Sigma, St. Louis, MO, USA), 5 μg/mL hydrocortisone (Sigma, St. Louis, MO, USA), and 2% penicillin/streptomycin (Gibco). To optimize the lactation state of the BuMECs, we determined the optimal prolactin (Sigma, St. Louis, MO, USA, Cas number: 9002-62-4) concentration to be 3 μg/mL, as shown in Figure S1. The cells were cultured in the aforementioned medium for 24 h until they reached approximately 80% confluence. For transfection, BuMECs were transiently transfected with 4 μg of pEGFP-N1-LEP plasmid using a 3:1 ratio of TransIntro^®^ EL Transfection Reagent (TransGen Biotech, Beijing, China), according to the manufacturer’s instructions. Total RNA extraction from BuMECs was performed using a method similar to that described in Section 2.2.

### 2.6. Gene Expression Detection and Analysis

Real-time quantitative PCR (RT-qPCR) primers were meticulously designed based on the *LEP* CDS obtained in this study (Table S2). To elucidate the role of *LEP* in milk fat synthesis, we assessed the expression of key genes involved in the JAK2-STAT3 and mTOR signaling pathways, both of which are critical for lipid metabolism. These genes included janus kinase 2 (*JAK2*), signal transducer and activator of transcription 3 (*STAT3*), suppressor of cytokine signaling 3 (*SOCS3*), phosphoinositide 3 kinase (*PI3K*), mammalian target of rapamycin (*mTOR*), and protein kinase B (*AKT1*). Additionally, we evaluated the expression of genes related to the fatty acid synthesis, desaturation, and transcriptional regulation, such as acetyl-coenzyme A carboxylase alpha (*ACACA*), fatty acid synthase (*FASN*), stearoyl-CoA desaturase (*SCD*), sterol-regulatory element-binding transcription factor 1 (*SREBF1*), and peroxisome proliferator-activated receptor-γ 1 (*PPARG1*).

RT-qPCR was performed using SYBR qPCR SuperMix Plus (dib) according to the manufacturer’s instructions on the CFX Connect Real-Time System (Bio-Rad). To ensure the accuracy and reliability of gene expression measurements, we used *β*-Actin (*ACTB*), glyceraldehyde-3-phosphate dehydrogenase (*GAPDH*), and *18S rRNA* as reference genes. Gene expression levels were calculated using the 2^−ΔΔCt^ method, and the geometric mean of the three 2^−ΔΔCt^ values for each gene was computed to determine the expression level [34,36,37]. Each sample was analyzed in triplicate, and the amplification efficiency of each gene was assessed using the LinRegPCR program (see Table S2).

### 2.7. TG Test in BuMECs

To quantify cellular TG, extraction was performed following the manufacturer’s instructions for the GPO-Trinder Triglyceride Assay Kit (Applygen Technologies, Beijing, China). Measurement and normalization of TG concentration were conducted using a Microplate Reader (Thermo Fisher Scientific) and a BCA Protein Assay Kit (Thermo Fisher Scientific), based on a linear regression of the standard curve.

### 2.8. Detection and Analysis of LEP Polymorphisms

Genomic DNA was isolated from blood samples using the phenol and chloroform method [38]. Primers for SNP detection were designed based on the buffalo *LEP* genome sequence NC_059164.1 (Table S2). The PCR reaction mixture consisted of 20 µL, containing 2 µL 10× PCR buffer, 0.4 µL of 10 pmol/µL forward and reverse primers, 0.16 µL of 25 mmol/L dNTP, 0.2 µL of 5 U/µL r-Taq, 2 µL of 50 ng/µL DNA templates, and water to reach a final volume of 20 µL. The PCR program included initial denaturation at 95 °C for 5 min, followed by 35 cycles of denaturation at 94 °C for 35 s, annealing at 57.5 °C for 40 s (with variations depending on the primer pairs), extension at 72 °C for 40 s, and a final extension at 72 °C for 5 min. PCR products were detected using agarose gel electrophoresis and bidirectionally sequenced by Shanghai Biotech Co., Ltd. (Shanghai, China).

### 2.9. Data Statistics

Differential expression and overexpression of the *LEP* gene were analyzed using three biological replicates, with results expressed as means ± standard error of the means (means ± SEM) for each experimental group. Statistical analyses and visualization were conducted using GraphPad Prism 8 software (GraphPad Software Inc., La Jolla, CA, USA). The statistical significance between the two groups was evaluated using the two-tailed Student’s *t*-test. For multiple group comparisons, a one-way ANOVA followed by the LSD test was applied. The *p*-values less than 0.05 were considered statistically significant, with additional thresholds set at *p* < 0.01 for 1% significance and *p* < 0.001 for 0.1% significance.

## 3. Results

### 3.1. Cloning and Identification of Buffalo LEP

**Figure 1:**
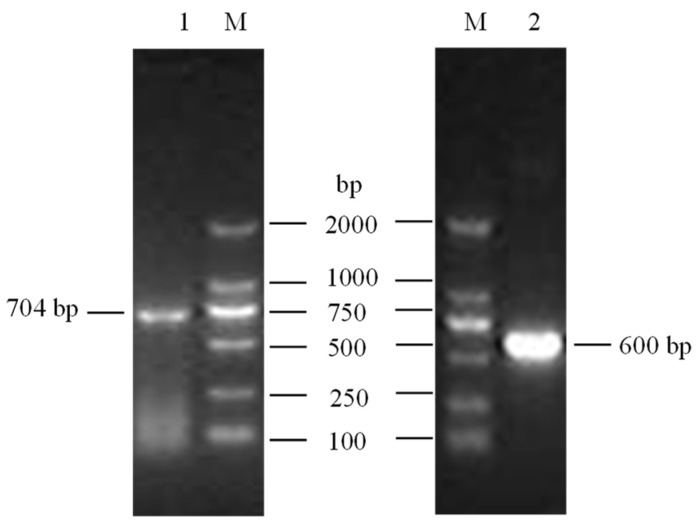
PCR results of clones for buffalo *LEP*_X1_pMD18-T (704 bp) and *LEP*_X2_pMD18-T (600 bp). M, Marker-DL2000; 1, *LEP*_X1_pMD18-T; 2, *LEP*_X2_pMD18-T. The original electrophoretic gels were in Figure S2.

**Figure 2:**
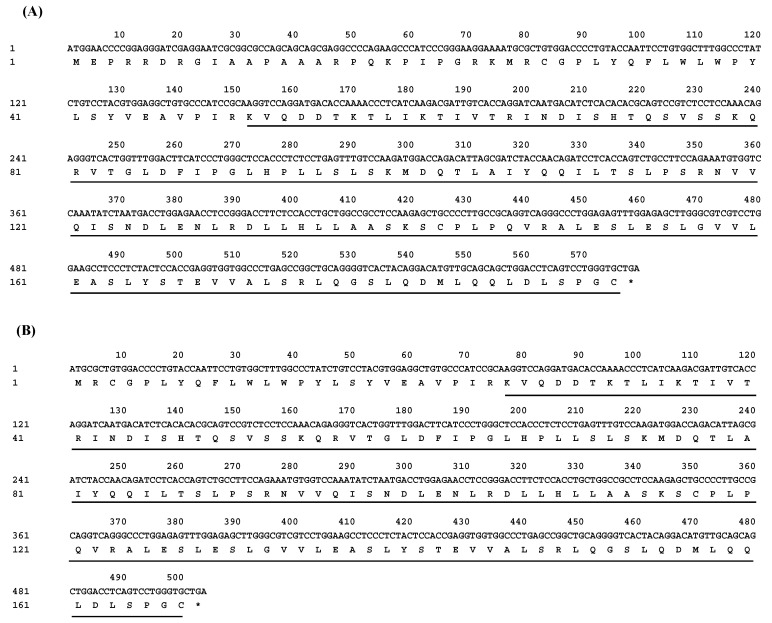
Buffalo *LEP*_X1 (**A**) and *LEP*_X2 (**B**) CDS obtained in this study and their deduced amino acid sequences. The amino acid sequence is located below the nucleotide sequence. The leptin-conserved domain region is underlined. The stop codon is indicated by an asterisk (*).

Two transcript variants of the *LEP* gene were successfully cloned from both lactating and non-lactating buffalo mammary gland tissues. (Figure 1 and Figure S2). The CDS was identified using ORF Finder, and a subsequent homology search through the BLAST program in the NCBI database confirmed that these CDSs share a high degree of identity (>97.00%) with *LEP* sequences from other species within the Bovidae family. The two transcript variants, designated as *LEP*_X1_CDS (XM_044946369.2, 579 bp) and *LEP*_X2_CDS (NM_001290901.1, 504 bp), encode protein variants consisting of 167 and 192 amino acid residues, respectively (Figure 2). Nucleotide composition analysis of buffalo *LEP*_X1_CDS revealed 21.42% A, 26.77% G, 19.86% T, and 31.95% C, resulting in an A+T content of 41.28% and a C+G content of 58.72%. For *LEP*_X2_CDS, the composition was 20.44% A, 25.40% G, 22.02% T, and 32.14% C, leading to an A+T content of 42.46% and a C+G content of 57.54%.

### 3.2. Transcriptional Region Structure of LEP

**Figure 3:**
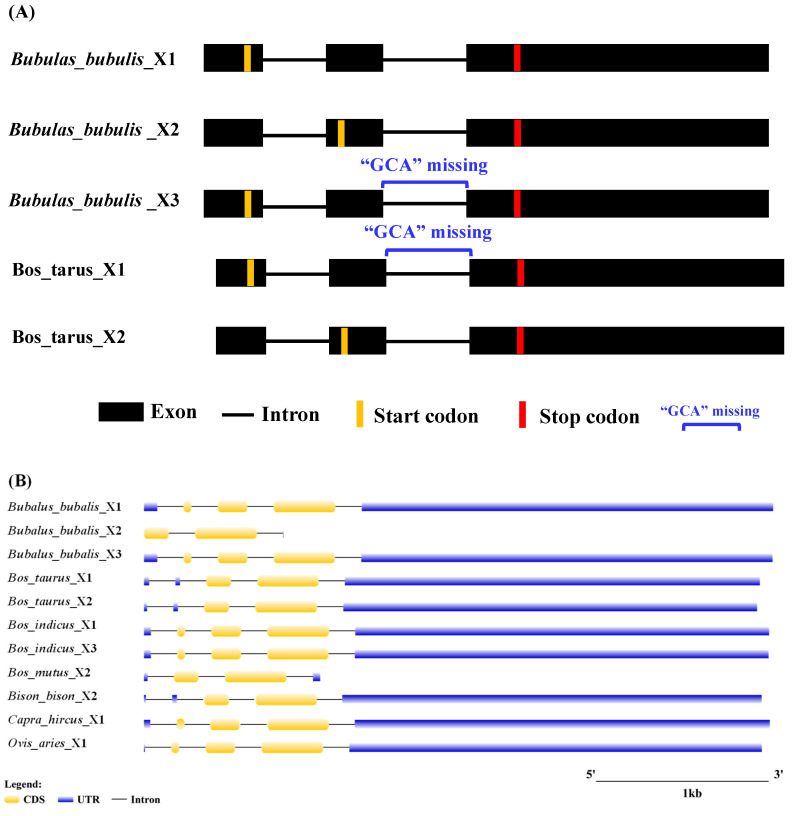
Schematic representation of structural differences in *LEP* transcript variants between *Bubalus bubalis* and *Bos taurus* (**A**) and transcriptional region structure (**B**).

The transcriptional region structures of the buffalo *LEP* were compared with those of other Bovidae species using data from the NCBI database (Table S3). In buffalo, three transcript variants were identified: *LEP*_X2 (504 bp), *LEP*_X1 (579 bp), and *LEP*_X3 (576 bp). *LEP*_X1 and *LEP*_X3 exhibit a three-nucleotide difference in exons 2–3 (Figure 3A). Analysis of transcriptional region structures revealed that the *LEP*_X2 variant is also present in yaks, bison, rats, and mice. Notably, the 5′UTR of *LEP*_X2 is absent in the buffalo sequences available in the NCBI database (Figure 3B). This structural variation highlights the evolutionary conservation and divergence among different species within the Bovidae family.

### 3.3. Identity of Amino Acid Sequences and Phylogenetic Tree of LEP

**Figure 4:**
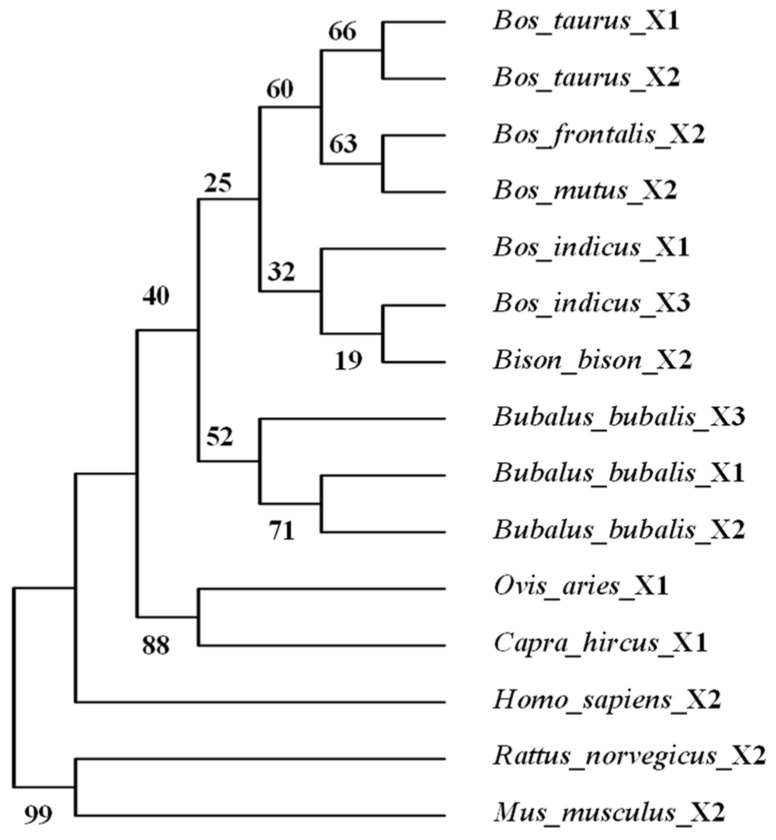
Phylogenetic tree of LEP in Bovidae and non-Bovidae species.

To explore the identity of LEP amino acid sequences between buffalo and other Bovidae species, several sequences were downloaded from the NCBI database (Table S1). The analysis showed that the identity of amino acid sequences between buffalo and other Bovidae species ranges from 97.6% to 99.5%. In contrast, the identity with non-Bovidae species ranges from 82.5% to 93.4% (Figure S3). Additionally, a phylogenetic tree was constructed based on amino acid sequences of LEP, which revealed that buffaloes were independently clustered into a distinct branch (Figure 4). This indicates a high level of conservation within the Bovidae family, while also highlighting the evolutionary divergence of buffaloes from other species.

### 3.4. Structures and Physicochemical Characteristics of LEP Protein

**Figure 5:**
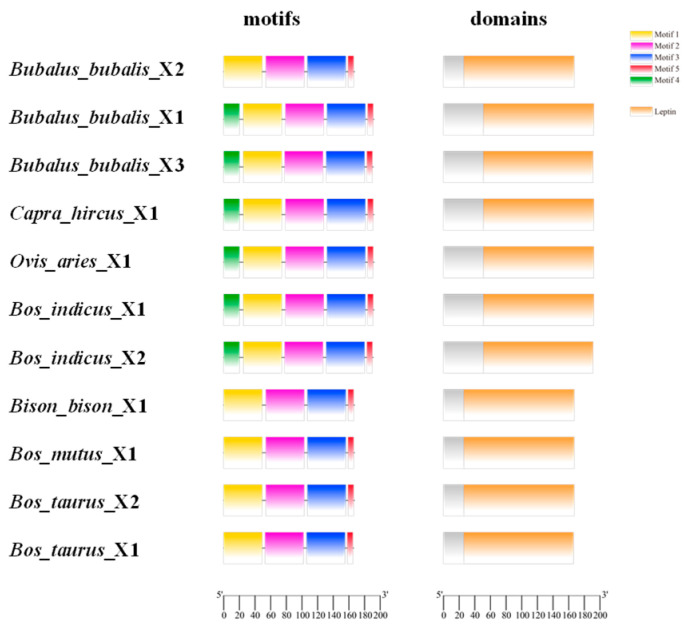
Motifs and conserved domains of LEP in Bovidae species.

The LEP protein contained a conserved Leptin domain comprising five conserved motifs, designated as motif 1–motif 5. These motifs were similarly present in cattle, zebu, sheep, and goats, while bison, yaks, rats, and mice exhibit four motifs due to differences in the 5’UTR (Figure 5). The secondary structure prediction for buffalo LEP_X1 revealed it was composed of 48.96% α-helix (94 AA), 40.62% random coils (78 AA), 5.73% extended chains (11 AA), and 4.69% β turn (9 AA) (Figure S4A). LEP_X2 comprised 56.29% α-helix (94 AA), 32.34% random coils (54 AA), 5.39% extended chains (9 AA), and 5.99% β turn (10 AA) (Figure S4B). LEP_X3 consisted of 56.02% α-helix (93 AA), 32.53% random coils (54 AA), 5.42% extended chains (9 AA), and 6.02% β turn (10 AA) (Figure S4C).

The tertiary structures of LEP proteins from buffaloes, cattle, zebu, yaks, sheep, and goats were constructed using homology modeling methods. The results showed that the coverage and identity were beyond 76% and 86.99% based on 1ax8.1. A model, respectively (Figure S5).

To elucidate the physicochemical characteristics of buffalo LEP proteins, comparisons were conducted between buffalo and other Bovidae species (Table S4). LEP_X2 exhibits distinct isoelectric points (<7) and higher hydrophilicity (hydrophilicity > 0) compared to LEP_X1 and LEP_X3 across Bovidae species.

### 3.5. Signal Peptide and Transmembrane Region

**Figure 6:**
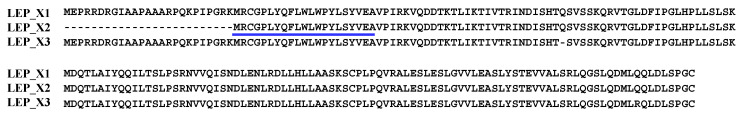
Signal peptide of buffalo LEP.

The prediction identified a signal peptide in buffalo LEP_X2 (1–21 aa, blue underlined). In contrast, no signal peptides were detected in buffalo LEP_X1 and LEP_X3 (Figure 6).

### 3.6. Biological Process, Molecular Function, and Functional Modification Site

Buffalo LEP participates in signal transduction processes (GO:0007165) and exhibits hormonal activity (GO:0005179). In this study, three types of functional modification sites were predicted in buffalo LEP_X1, including acetylation sites, Casein Kinase II (CK2) phosphorylation sites, and Protein Kinase C (PKC) phosphorylation sites (Table S5). However, LEP_X2 contains two types of active sites, CK2 and PKC, suggesting potential divergent effects of LEP_X1 and LEP_X2 on milk fat synthesis.

### 3.7. Differential Expression of LEP

**Figure 7:**
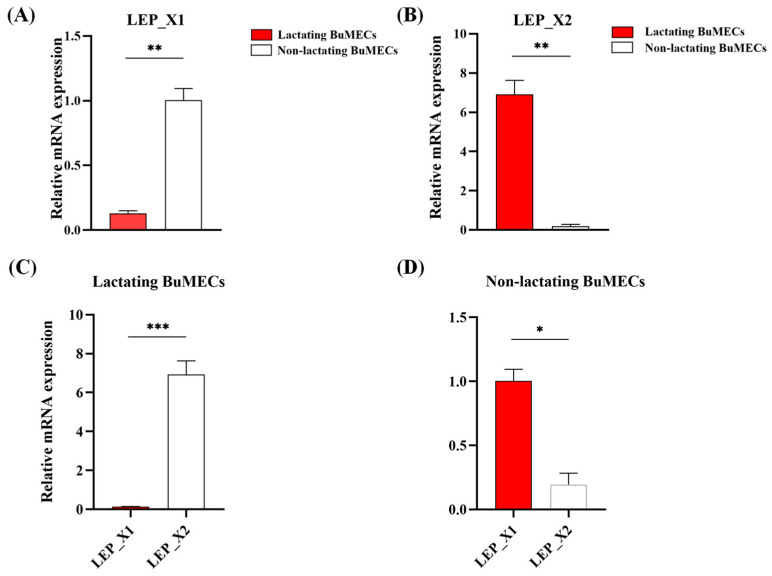
Differential expression of *LEP*_X1 (**A**), *LEP*_X2 (**B**), along with *LEP* expression in lactating (**C**) and non-lactating (**D**) BuMECs. Values are presented as means ± SEM; * *p* < 0.05, ** *p* < 0.01, *** *p* < 0.001.

To optimize lactation conditions in BuMECs, we supplemented the cell culture with 3 μg/mL of prolactin (Figure S1). Experimental and control groups were established, with and without prolactin, respectively, and the expression of *LEP*_X1 and *LEP*_X2 was assessed using qPCR. Results revealed a significant decrease in *LEP*_X1 expression in lactating compared to non-lactating BuMECs (*p* < 0.01, Figure 7A), whereas *LEP*_X2 expression was significantly elevated in lactating BuMECs (*p* < 0.01, Figure 7B). Interestingly, *LEP*_X2 expression was notably higher than *LEP*_X1 in lactating BuMECs (*p* < 0.001, Figure 7C), while it was significantly lower in non-lactating BuMECs (*p* < 0.05, Figure 7D). These findings suggest a predominant role of *LEP*_X2 in lactating BuMECs, whereas *LEP*_X1 may primarily function in non-lactating BuMECs.

### 3.8. Overexpression of LEP_X1 Upregulates Genes Related to Milk Fat Synthesis in BuMECs and Increases TG Concentration

**Figure 8:**
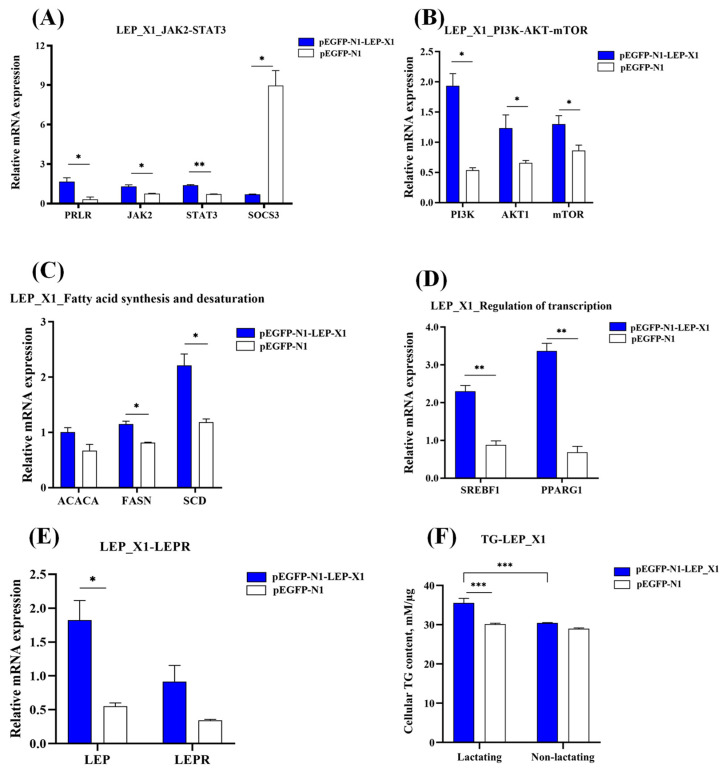
Effect of *LEP*_X1 overexpression on milk fat synthesis in BuMECs with PRL. Changes are shown for the JAK2-STAT3 signaling pathway (**A**), the mTOR signaling pathway (**B**), fatty acid synthesis and desaturation (**C**), regulation of transcription (**D**), LEPR expression (**E**), and cellular TG concentration (**F**). Data are presented as means ± SEM for three individual cultures; * *p* < 0.05, ** *p* < 0.01, *** *p* < 0.001.

To explore the effect of *LEP*_X1 on milk fat synthesis, the overexpression vector pEGFP-N1-LEP_X1 was transfected into BuMECs. We then examined changes in the expression of genes related to milk fat synthesis within JAK2-STAT3 (*JAK2*, *STAT3*, and *SOCS3*) and mTOR (*PI3K*, *AKT1*, and *mTOR*) signaling pathways, fatty acid synthesis and desaturation (*ACACA*, *FASN*, and *SCD*), and transcriptional regulation (*SREBF1* and *PPARG*) processes. Compared to the negative control (NC, pEGFP-N1), *LEP*_X1 overexpression significantly increased the expression of *PRLR* (*p* < 0.05), *JAK2* (*p* < 0.05), and *STAT3* (*p* < 0.01) while decreasing *SOCS3* (*p* < 0.05) (Figure 8A). Additionally, it significantly upregulated *PI3K* (*p* < 0.05), *AKT1* (*p* < 0.05), and *mTOR* (*p* < 0.05) (Figure 8B), as well as *FASN* (*p* < 0.05), *SCD* (*p* < 0.05) (Figure 8C), and *SREBF1* (*p* < 0.01), and *PPARG* (*p* < 0.01) (Figure 8D). This was also accompanied by an increase in cellular TG (*p* < 0.001, Figure 8F) in lactating BuMECs. However, *ACACA* (*p* > 0.05, Figure 8C) and *LEPR* (*p* > 0.05, Figure 8E) expression levels were unaffected.

### 3.9. Overexpression of LEP_X2 Upregulates the Genes Related to Milk Fat Synthesis in BuMECs and Increases TG Concentration

**Figure 9:**
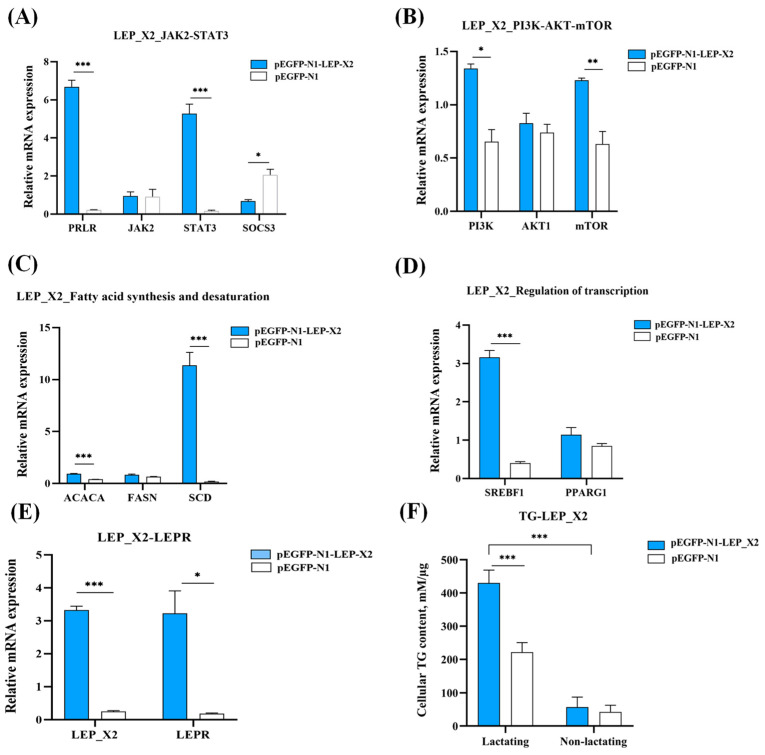
Effects of *LEP*_X2 overexpression on milk fat synthesis in BuMECs with PRL. Changes are shown for the JAK2-STAT3 signaling pathway (**A**), the mTOR signaling pathway (**B**), fatty acid synthesis and desaturation (**C**), regulation of transcription (**D**), *LEPR* expression (**E**), and cellular TG concentration (**F**). Data are presented as means ± SEM for three individual cultures; * *p* < 0.05, ** *p* < 0.01, *** *p* < 0.001.

We examined the effect of *LEP*_X2 overexpression on the expression of genes related to milk fat synthesis and cellular TG concentration in BuMECs. Compared to the NC, *LEP*_X2 overexpression significantly increased the expression of *PRLR* (*p* < 0.001) and *STAT3* (*p* < 0.001), while decreasing *SOCS3* (*p* < 0.05) (Figure 9A). It also significantly upregulated *PI3K* (*p* < 0.05) and *mTOR* (*p* < 0.01) expression (Figure 9B). Furthermore, *LEP*_X2 overexpression led to significant increases in *ACACA* (*p* < 0.001) and *SCD* (*p* < 0.001) (Figure 9C), *SREBF1* (*p* < 0.001, Figure 9D), and *LEPR* (*p* < 0.05, Figure 9E), accompanied by an increase in cellular TG concentration (*p* < 0.001, Figure 9F). In contrast, *JAK2* (*p* > 0.05, Figure 9A), *AKT1* (*p* > 0.05, Figure 9B), *FASN* (*p* > 0.05, Figure 9C), and *PPARG1* (*p* > 0.01, Figure 9D) expression levels were unaffected.

### 3.10. Antagonism Interaction between LEP_X1 and LEP_X2

**Figure 10:**
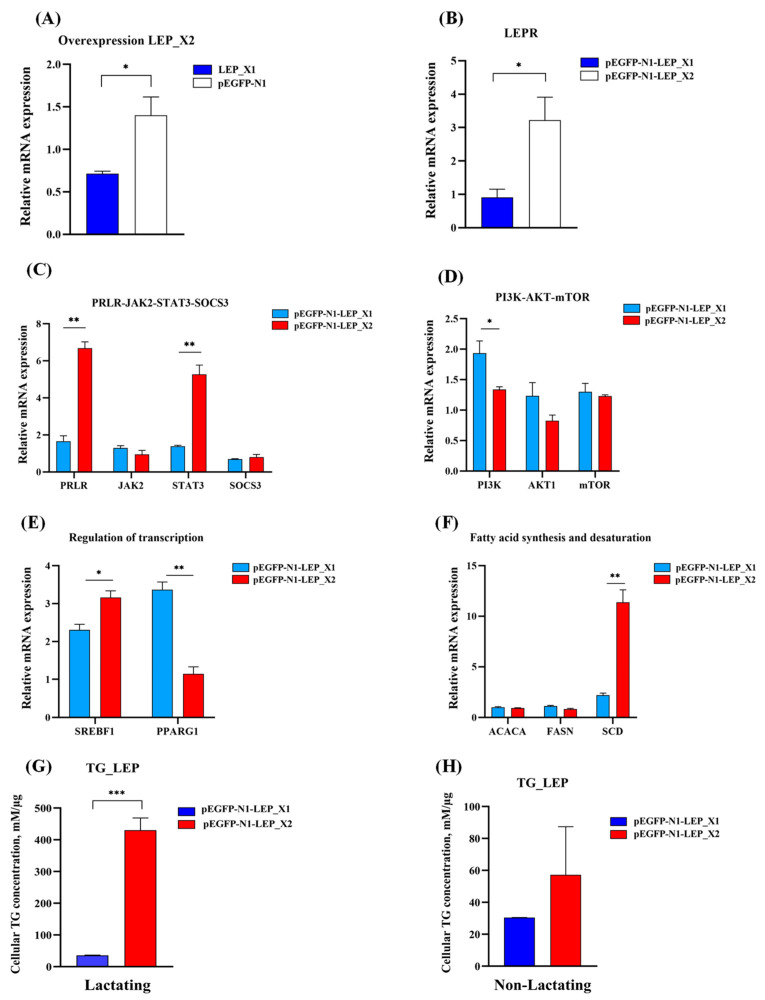
Comparison of gene expression related to milk fat synthesis following *LEP*_X1 and *LEP*_X2 overexpression. The expression levels of *LEP*_X1 after overexpression of *LEP*_X2 (**A**), *LEPR* (**B**), genes in the JAK2-STAT3 signaling pathway (**C**), the mTOR signaling pathway (**D**), regulation of transcription (**E**), fatty acid synthesis and desaturation (**F**), and cellular TG concentration in lactating (**G**) and non-lactating (**H**). Data are presented as means ± SEM for three individual cultures; * *p* < 0.05, ** *p* < 0.01, *** *p* < 0.001.

The overexpression results for *LEP*_X1 and *LEP*_X2 revealed that both upregulated the expression of genes related to milk fat synthesis, indicating their positive regulation of this process. However, *LEP*_X2 overexpression suppressed *LEP*_X1 expression (Figure 10A), suggesting an antagonistic effect between them. Compared to *LEP*_X1 overexpression, *LEP*_X2 overexpression resulted in higher expression levels of several genes, including *LEPR* (*p* < 0.05, Figure 10B), *PRLR* (*p* < 0.01), and *STAT3* (*p* < 0.01) (Figure 10C), *SREBF1* (*p* < 0.05, Figure 10E), and *SCD* (*p* < 0.01, Figure 10F), as well as increased cellular TG concentration in lactating BuMECs (*p* < 0.001, Figure 10G). Conversely, the expression of *PI3K* (*p* < 0.01, Figure 10D) and *PPARG1* (*p* < 0.01, Figure 10F) was significantly decreased.

### 3.11. Population Variation Analysis

Four SNPs were identified in this study: c.148C>T, c.435G>A, c.450G>A, and c.558G>A. Of these, only c.148C>T was a non-synonymous substitution (p.Arg50Cys) and was only present in swamp buffalo, whereas the other SNPs were synonymous and were present in both buffalo types. The c.148C>T was predicted to have deleterious effects on LEP protein (score = 0.707) and decreased LEP protein stability (ΔΔG = −0.7635 Kcal/mol, indicating decreased stability; prediction accessed from http://mupro.proteomics.ics.uci.edu, last accessed on 10 February 2024). However, no new functional active sites were predicted at c.148T (Table S5). Further analysis indicated that the SNPs found in buffalo *LEP* did not significantly affect codon bias usage, suggesting that these SNPs are unlikely to have a substantial impact on the function of the LEP as a result of changing the frequency of codon usage (Tables S6 and S7).

Genotype and allele frequency calculations indicated that c.558G and c.450G were the frequency-dominant alleles for river and swamp buffalo, respectively (Table 1). The Hardy–Weinberg equilibrium test showed that both SNP435 and SNP450 were in disequilibrium in swamp buffalo (*p* < 0.05).

### 3.12. Sequence Differences Among Bovidae Species

Five major haplotypes were identified based on polymorphisms in buffalo *LEP* CDS (Table S8), with additional haplotypes listed in Table S9. Notably, the haplotypes CGAA and TGGG were unique to swamp buffaloes. The sequences of the five major haplotypes had been submitted to the NCBI database with the accession numbers KP864436.2-KP864440.2 for nucleotide sequences and ALA50393.2-ALA50397.2 for amino acid sequences.

**Figure 11:**
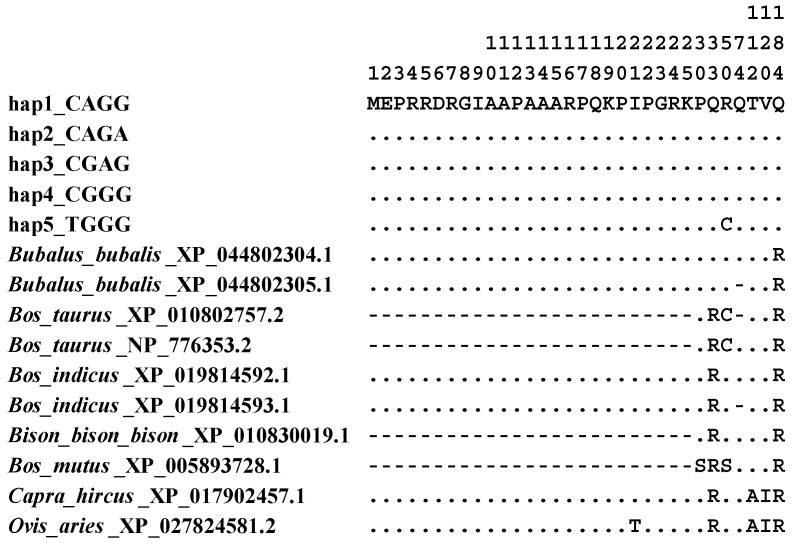
Differences in LEP amino acid sequences among Bovidae species. Numbers represent positions. Different letters represent different amino acids. Dots (⋅) indicate identity with hap1_CAGG. Horizontal bars (-) indicate amino acid deletions.

To explore the sequence differences between buffalo and other Bovidae species, we conducted a comparative analysis of haplotype sequences obtained by this study with homologous sequences retrieved from the NCBI database (Figure 11 and Figure S6). The results showed that the *LEP* CDS in buffalo (NM_001290901.1), cattle (NM_173928.2), bison, and yak exhibited a 75 bp reduction in length compared to other Bovidae species aligned with the Hap1 (used as reference sequence here, KP864436.2). In contrast, the *LEP* CDS of buffalo (XM_044946370.2) and zebu (XM_019959034.1) displayed a reduction of only 3 bp (Figure S6). Nucleotide sequence analysis showed that nucleotide differences existed between Bovidae species at the c.98 and c.456 loci (with the c.98 locus leading to corresponding changes in the encoded amino acids), which could be used as specific molecular markers to differentiate buffalo from other Bovidae species.

## 4. Discussion

Buffalo milk is emerging as a significant economic source globally. Understanding the compositions and concentrations of proteins, fats, lactose, vitamins, and minerals in buffalo milk is essential for producing healthier and tastier dairy products [39]. In cattle, leptin was detected in the mammary gland, where it stimulates the synthesis of milk lipids and proteins and coordinates the expression of genes related to fatty acid synthesis [40,41]. However, the precise molecular mechanism of *LEP* on milk fat synthesis has not been well elaborated in buffaloes.

In this study, we identified and characterized two *LEP* transcript variants, *LEP_X1* and *LEP_X2*, from buffalo mammary gland tissues. The CDSs of *LEP*_X1 and *LEP*_X2 are 579 bp and 504 bp, encoding proteins of 192 and 167 amino acid residues, respectively. Both variants share a Leptin domain, essential for binding to the leptin receptor (LEPR) and activating key signaling pathways that regulate mammary epithelial cells [41]. Notably, LEP_X1 lacks a signal peptide, suggesting it functions mainly through paracrine mechanisms, while LEP_X2, which has a signal peptide, likely operates through both autocrine and paracrine mechanisms. Despite a 99.4% identity in their amino acid sequences, LEP_X1 is more structurally flexible and hydrophilic, whereas LEP_X2 is hydrophobic, leading to potential functional differences [42].

Milk fat synthesis is regulated by numerous cytokines, hormones, and downstream signaling pathways [43].Gene Ontology (GO) enrichment analysis indicates that both LEP_X1 and LEP_X2 play important roles in the JAK2-STAT3 and adipokine signaling pathways and are involved in the positive regulation of the PI3K-AKT-mTOR pathway, which is consistent with the analysis of protein interactions with LEP (Figure S7). LEPR, a member of the class I cytokine receptor family, mediates LEP’s activation of the JAK-STATs signaling pathway, with JAK2 and STAT3 being key components essential for LEP signaling [44]. Genes in the JAK-STAT signaling pathway are involved in immune response, inflammation, and lipid metabolism [45,46]. In this pathway, SOCS proteins, particularly SOCS3, provide negative feedback by inhibiting the activation and phosphorylation of JAKs and STATs [47]. CEBPA, a member of the C/EBPs family, is essential for adipocyte differentiation and lipid metabolism [48]. Furthermore, PPARG, a member of the PPARs family, promotes transcription of genes related to lipid synthesis within the JAK2-STAT3 signaling pathway [49]. The mTOR signaling pathway is also closely associated with milk fat synthesis. The LEP-LEPR signaling pathway activates PI3K, AKT1, and mTOR expression, stimulating downstream pathways involved in milk lipid synthesis. This activation promotes mTORC1’s activity, maintains the cleavage activity of SREBP-1, facilitates translation, and triggers a positive feedback loop involving CEBPA and PPARG, further enhancing lipid synthesis [50,51].

Our data indicate that expression of *LEP*_X1 and *LEP*_X2 is higher in lactating BuMECs compared to non-lactating BuMECs. However, *LEP*_X2 expression is notably higher in lactating BuMECs, suggesting a major role for *LEP*_X2 in lactation. In contrast, *LEP*_X1 expression is higher in non-lactating BuMECs, indicating that *LEP*_X1 may be involved in the proliferation of BuMECs [52]. *LEP*_X2 has been investigated as a major transcript variant in recent studies, with its expression detected in several mammals, including humans, pandas, and mithun, underscoring its significance across species [53,54,55].

To further explore the function of two transcript variants, we conducted functional experiments at cellular levels. Our results demonstrated overexpression of *LEP*_X1 and *LEP*_X2 significantly influenced the expression of genes related to milk fat synthesis. *LEP*_X1 overexpression notably enhanced the expression of genes in the JAK2-STAT3 and PI3K-AKT-mTOR signaling pathways (*LEPR*, *JAK2*, *STAT3*, *PI3K*, *AKT1,* and *mTOR*) and increased the expression of *FASN*, *SCD*, *SREBF1*, and *PPARG1*, which are related to fatty acid synthesis and desaturation. Similarly, overexpression of *LEP_X2* promoted the expression of *LEPR*, *PRLR*, *STAT3*, *PI3K*, *mTOR*, *ACACA*, *SCD,* and *SREBF1*. These findings suggest that both *LEP_X1* and *LEP_X2* positively impact milk fat synthesis through the JAK2-STAT3 and mTOR signaling pathways, with *LEP_X2* having a more pronounced effect, as indicated by greater upregulation of milk fat synthesis-related genes and a significant increase in cellular triglyceride (TG) concentration.

Compared to *LEP*_X1 overexpression, *LEP*_X2 overexpression resulted in a 3.52-fold, 4.02-fold, 3.79-fold, 5.13-fold, and 1.37-fold increase in the expression levels of *LEPR*, *PRLR*, *STAT3*, *SCD,* and *SREBF1*, respectively, as well as a remarkable 12.10-fold increase in cellular TG concentration. However, *LEP*_X2 overexpression inhibited *LEP*_X1 expression, suggesting an antagonistic relationship between them. In lactating BuMECs, the higher expression of *LEP*_X2 compared to *LEP*_X1 suggests that *LEP*_X2 plays a dominant role in promoting milk fat synthesis. Since LEP_X1 lacks a signal peptide, it likely binds to LEPR through a paracrine mechanism [56], while LEP_X2, which possesses a signal peptide, likely binds to LEPR through both autocrine and paracrine mechanisms. LEPR activation relies on two key steps: JAK2 activation and STAT3 recruitment [57]. Compared to the changes in gene expression following *LEP_X1* overexpression, *LEP*_X2 overexpression resulted in higher *LEPR* expression, indicating that *LEP*_X2 binds to LEPR more preferentially and efficiently. The higher expression of *LEPR*, *STAT3*, and *SREBF1* after *LEP_X2* overexpression suggests that *LEP*_X2 more efficiently binds to LEPR, primarily promoting milk fat synthesis-related gene expression via *STAT3* and *SREBF1* regulation. Conversely, higher expression of *PI3K* and *PPARG1* was higher after overexpression of *LEP*_X1 compared to *LEP*_X2 overexpression, suggesting that *LEP*_X1 positively regulates milk fat synthesis-related genes via PPARG1. It is important to note that LEP regulation in the mammary gland is more complex than in BuMECs, as LEP is produced by both the mammary fat pad and mammary epithelial cells [58], contributing to the maintenance and regulation of epithelial cells [41].

Recent studies have shown that SNPs in the *LEP* gene are associated with milk production traits such as milk yield and composition in various Bovidae species [4,6,59]. For instance, in Holstein cows, the c.73C>T (p.Arg25Cys) has been linked to milk yield, with cows carrying the TT genotype producing more milk than those with the CC genotype [59]. In this study, we identified four substitutions in the *LEP* CDS in buffalo. Among these, c.148C>T (p.Arg50Cys) is a nonsynonymous substitution, while the other three are synonymous. The c.148T allele is potentially deleterious, affecting the stability of the LEP protein. Furthermore, the c.435G and c.450G alleles were found in river and swamp buffalo. The Hardy–Weinberg equilibrium test showed that SNP435 and SNP450 were not in equilibrium in swamp buffalo (*p* < 0.05). A comparison of nucleotide sequences revealed that the CDS length of buffalo *LEP*_X1 was 75 bp longer than that of bison and yak. Additionally, c.98A and c.456T variations in *LEP* CDS distinguish buffalo from other Bovidae species, providing molecular markers for species identification.

## 5. Conclusions

Our results indicate that *LEP*_X1 primarily promotes milk fat synthesis through the regulation of the PI3K-AKT1-mTOR and JAK2-STAT3 signaling pathways and PPARG1. *LEP*_X2 drives milk fat synthesis in BuMECs through the regulation of the JAK2-STAT3 and PI3K-AKT1-mTOR signaling pathways and SREBF1. The down-regulation of *LEP_X1* due to *LEP_X2* overexpression suggests an antagonistic relationship between the two transcript variants, with *LEP*_X2 playing a dominant role in milk fat synthesis through a STAT3-mediated recruitment process and its high affinity for LEPR. Our study provides valuable insights into the structure and function of the transcriptional variants of the buffalo *LEP* gene, especially their functional role in milk fat synthesis.

## Figures and Tables

**Table 1:** Population genetic information of SNP loci in the *LEP* gene.

Population	SNP	Genotype	Genotype Frequency (%)	Allele	Allele Frequency (%)	*p*-Value ^1^
River buffalo	c.148C>T	CC	1.0000	C T	1.0000 0.0000	−
		CT	0.0000			
		TT	0.0000			
	c.435G>A	AA	0.0417	A G	0.1667 0.8333	0.53751
		AG	0.2500			
		GG	0.7083			
	c.450G>A	AA	0.0417	A G	0.2500 0.7500	0.65497
		AG	0.4167			
		GG	0.5416			
	c.558G>A	AA	0.0417	A G	0.1042 0.8958	0.06186
		AG	0.1250			
		GG	0.8333			
Swamp buffalo	c.148C>T	CC	0.9756	C T	0.9878 0.0122	1.00000
		CT	0.0244			
		TT	0.0000			
	c.435G>A	AA	0.4634	A G	0.5610 0.4390	0.00008
		AG	0.1951			
		GG	0.3415			
	c.450G>A	AA	0.0732	A G	0.1707 0.8293	0.03474
		AG	0.1951			
		GG	0.7317			
	c.558G>A	AA	0.0976	A G	0.2195 0.7805	0.05150
		AG	0.2439			
		GG	0.6585			

^1^ *p*-value of the Hardy–Weinberg equilibrium test.

## Data Availability

The data analyzed during the current study are available from the corresponding authors upon reasonable request.

## Supplementary Materials

The following supporting information can be downloaded at: https://www.mdpi.com/article/10.3390/ani14162446/s1. Figure S1: TG content measured across varying concentrations of prolactin; Figure S2: Original electrophoretic gels displaying the PCR results of buffalo *LEP*_X1_pMD18-T (A) and *LEP*_X2_pMD18-T (B) clones, with respective lengths of 704 bp and 600 bp. M, Marker-DL2000; lanes 1–24: clones of *LEP*_X1_pMD18-T (A); lanes 1–10: clones of *LEP*_X2_pMD18-T (B). The skewed lines observed in the electrophoretic gels are attributed to misaligned electrodes within the electrophoresis bath, while the smearing lines are indicative of false-positive clones; Figure S3: Percent identity/divergence amino acid sequences of LEP between Bovidae and non-Bovidae species; Figure S4: Predicted secondary structure of buffalo LEP_X1 (A), LEP_X2 (B), and LEP_X3 (C). Alpha helices, β turns, extended strands, and random coils are indicated with h, t, e, and c, respectively; Figure S5: Comparative analysis of the tertiary structures of LEP variants across different species: LEP_X1, LEP_X2, LEP_X3 in buffalo (a–c), LEP_X1, LEP_X2 in cattle (d,e), LEP_X1, LEP_X2 in zebu (f,g), LEP_X2 in bison (h) and yak (i), and LEP_X1 in goat (j) and sheep (k); Figure S6: Nucleotide sequence differences in LEP among Bovidae species; Figure S7: Protein interactions network involving LEP in buffalo; Table S1: Sequence information of the *LEP* gene retrieved from the NCBI database; Table S2: Primer sequences used for PCR and RT-qPCR; Table S3: Structural details of the transcriptional regions of the *LEP* gene; Table S4: Physicochemical characteristics of the LEP protein; Table S5: Predicted functional active sites within the buffalo LEP protein; Table S6: Effective number of codons (ENc), GC content, and GC3s content in *LEP* gene haplotypes of buffalo; Table S7: Relative synonymous codon usage (RSCU) values for *LEP* gene haplotypes in buffalo; Table S8: Major haplotypes identified within the buffalo *LEP* gene; Table S9: Detailed haplotype information for the *LEP* gene in buffalo.
