# Supplementary material for: LEP Gene Promotes Milk Fat Synthesis via the JAK2-STAT3 and mTOR Signaling Pathways in Buffalo Mammary Epithelial Cells
Source: Animals (Basel). 2024 Aug 22;14(16):2446. doi: 10.3390/ani14162446 (PMC11350831; doi:10.3390/ani14162446)
Supplement: Supplementary file 1 [file animals-14-02446-s001.zip › Supplementary material_For proofead.pdf]

LEP gene promotes milk fat synthesis via the JAK2-STAT3 and mTOR signaling pathways in buffalo mammary epithelial cells

Ruixia Gao[1, †], Qunyao Zhu[1, †], Lige Huang[1], Xinyang Fan[1], Xiaohong Teng[1] and Yongwang Miao[1,*]

1 Institute of Animal Genetics and Breeding, College of Animal Science and Technology, Yunnan Agricultural University, Kunming 650201, Yunnan, China; malegreshell@126.com (R.G.); 18213524162@163.com (Q.Z.); 18838969693@163.com (L.H.); xinyangfan1@126.com (X.F.); 13698786782@163.com (X.T.)

* Correspondence: yongwangmiao1@126.com; Tel.: +86-137-0065-0615.

† These authors contributed equally to this work.

Supplementary materials

Table S1. Sequence information of *LEP* gene retrieved from the NCBI database.

Species	Accession number of nucleotide sequences	Accession number of protein sequences	CDS length (bp)	Coverage
Bubalus bubalis	NM_001290901.1	NP_001277830.1	504	exon2-3
Bubalus bubalis	XM_044946369.2	XP_044802304.1	579	exon1-3
Bubalus bubalis	XM_044946370.2	XP_044802305.1	576	exon1-3
Bos taurus	NM_173928.2	NP_776353.2	504	exon2-3
Bos taurus	XM_010804455.4	XP_010802757.2	501	exon2-3
Bos indicus	XM_019959033.1	XP_019814592.1	579	exon1-3
Bos indicus	XM_019959034.1	XP_019814593.1	576	exon1-3
Bison bison bison	XM_010831717.1	XP_010830019.1	504	exon2-3
Bos mutus	XM_005893666.1	XP_005893728.1	504	exon2-3
Bos frontalis	EU642566.1	ACF40216.1	504	exon2-3
Camelus bactrianus	XM_010947570.2	XP_010945872.2	579	exon1-3
Ceratotherium simum	XM_004418724.2	XP_004418781.2	570	exon1-3
Equus asinus	XM_014854289.2	XP_014709775.1	570	exon1-3
Capra hircus	XM_018046968.1	XP_017902457.1	579	exon1-3
Ovis aries	XM_027968780.2	XP_027824581.2	579	exon1-3
Sus scrofa	XM_021078502.1	XP_020934161.1	579	exon1-3
Rattus norvegicus	NM_013076.3	NP_037208.1	504	exon2-3
Mus musculus	NM_008493.3	NP_032519.1	504	exon2-3
Homo sapiens	NM_000230.3	NP_000221.1	504	exon2-3

Table S2. Primer sequences used for PCR and RT-qPCR.

	Primers (5′ to 3′)*	Product length (bp)	Annealing temperature (°C)	Efficiency**	Usage
LEP	F: AGTTGTGCTTCGGCGGCTAT<br>R: AGGATGCCCACATAGGCTCT	704	64.7	/	X1_CDS isolation and bidirectional sequencing
LEP	F: GAAGGAAAATGCGCTGTGGAC<br>R: AGGATGCCCACATAGGCTCT	704	61.6	/	X2_CDS isolation and bidirectional sequencing
LEP	F: TTGTGCTTCGGCGGCTATAA<br>R: GGCCAAAGCCACAGGAATTG	151	54.0	2.06	X1_Expression detection
LEP	F: ATGGACCAGACATTGGCGATC<br>R: CTCGGTGGAGTAGAGGGAGGC	208	54.0	2.08	X2_Expression detection
LEP	F: AAGCTTATGGAACCCCGGAGGGATCG (Hind III)<br>R: GAATTCGCACCCAGGACTGAGGTC (EcoR I)	576	61.7	/	Vector construction and bidirectional sequencing
LEP	F: AAGCTTATGCGCTGTGGACCCCTGTA (Hind III)<br>R: GAATTCGCACCCAGGACTGAGGTC (EcoR I)	501	67.2	/	Vector construction and bidirectional sequencing
Exon1	F: TGGCGCTGCGGGTGCGCCC<br>R: CTCCGCCGGTCTTTCTTGG	173	62.1	/	SNP detection and bidirectional sequencing
Exon2	F: CTTGATTCCGCCGCACCT<br>R: GCTTCCTCCGCCAACCTC	452	63.0	/	SNP detection and bidirectional sequencing
Exon3	F: GGGAAGGGCAGAAAGATAGG<br>R: CGGCTGAGAGGAGCGAG	695	56.0	/	SNP detection and bidirectional sequencing
LEPR	F: GAGTCACCTCTGCTCCCC<br>R: ATTTCCATACGCAAACCT	230	60.0	2.09	Expression detection
PPARG	F: GCTCCAAGAGTACCAAAGTG<br>R: GCTCCAAGAGTACCAAAGTG	204	53.7	2.01	Expression detection

Table S2 continued

	Primers (5′ to 3′)*	Product length (bp)	Annealing temperature (°C)	Efficiency**	Usage
SOCS3	F: CAGTCGGGGACCAAGAACC<br>R: CGAGGAGGGCGAGGAGGAG	189	53.3	2.11	Expression detection
PI3K	F: CAGTCACCTCTCAACCCA<br>R: GCCGTAAATCATCACCAT	160	60.0	1.90	Expression detection
JAK2	F: GATCTGGCAACAAGGAAT<br>R: CGCTGGTGGGCTTTTACT	252	60.0	2.02	Expression detection
STAT3	F: GCCATTAGTCATCAAGACCG<br>R: TCACTTTCGTGTTTGTGCC	176	60.0	2.05	Expression detection
mTOR	F: ATGCTGTCCCTGGTCCTTATG<br>R: GGGTCAGAGAGTGGCCTTCAA	178	60.0	2.10	Expression detection
AKT1	F: CCAAGTCCCTGCTCTCGG<br>R: TTCGCTGTCCACCCCCTC	266	60.0	1.95	Expression detection
ACTB	F: TGGGCATGGAATCCTG<br>R: GGCGCGATGATCTTGAT	196	60.0	2.03	Expression detection
FASN	F: AGGCCAGCTCCGAAGGCAACA<br>R: TACCACGTCGGCCACTTGTGTC	209	60.0	2.06	Expression detection
SCD	F: CGTGCCGTGGTATCTGTGG<br>R: AAAGGTGTGGTGGTAGTTGTGG	217	57.9	2.00	Expression detection
GAPDH	F: ATGGAGAAGGCTGGGGCTCA<br>R: GCAGGAGGCATTGCTGACAA	144	60.0	2.07	Expression detection
18S rRNA	F: GCGGCTTTGGTGACTCTA<br>R: CTGCCTTCCTTGGATGTG	195	60.0	1.95	Expression detection

Note: * Primer direction (F: forward; R: reverse); underlined letters indicate the restriction sites.

** Efficiency of amplification as calculated by LinRegPCR (www.linregpcr.nl).

Table S3. Structural details of the transcriptional regions of the *LEP* gene.

Species	Transcript variants	5′UTR (bp)	3′UTR (bp)	Exon1 (bp)	Exon2 (bp)	Exon3 (bp)	Coverage	CDS length (bp)
Bubalus bubalis	NM_001290901.1 (X2)	/	/	/	144	360	exon2-3	504
Bubalus bubalis	XM_044946369.2 (X1)	77	2366	124	172	2726	exon1-3	579
Bubalus bubalis	XM_044946370.2 (X3)	78	2366	125	172	2723	exon1-3	576
Bos taurus	NM_173928.2 (X2)	47	2379	19	172	2739	exon2-3	504
Bos taurus	XM_010804455.4 (X1)	59	2387	/	172	2744	exon1-3	501
Bos indicus	XM_019959033.1 (X1)	41	2380	88	172	2740	exon1-3	579
Bos indicus	XM_019959034.1 (X3)	41	2380	88	172	2737	exon1-3	576
Bos mutus	XM_005893666.1 (X2)	22	40	/	166	400	exon2-3	504
Bison bison bison	XM_010831717.1 (X2)	41	2412	13	172	2772	exon2-3	504
Capra hircus	XM_018046968.1 (X1)	38	2387	85	172	2747	exon1-3	579
Ovis aries	XM_027968780.2 (X1)	7	2372	54	172	2732	exon1-3	579
Rattus norvegicus	NM_013076.3 (X2)	48	2738	14	178	3098	exon2-3	504
Mus musculus	NM_008493.3 (X2)	59	2694	28	175	3054	exon2-3	504

Table S4. Physicochemical characteristics of the LEP protein.

Basic physical and chemical properties	Buffalo_X1	Buffalo_X2	Buffalo_X3	Cattle_X1	Cattle_X2	Zebu_X1	Zebu_X3	Yak_X2	Bison_X2	Sheep_X1	Goat_X1
Number of amino acids	192	167	191	166	167	192	191	167	167	192	192
Molecular weight (kDa)	21.38	18.69	21.28	18.53	18.66	21.44	21.31	18.64	18.72	21.41	21.42
Isoelectric point (pI)	9.07	6.82	9.27	6.81	6.81	9.43	9.43	6.82	7.73	9.43	9.43
Strongly acidic amino acid (D, E)	17	15	17	10	15	17	17	15	15	17	17
Strongly basic amino acid (K, R)	21	15	22	15	15	23	23	15	16	23	23
Polar amino acid (N, C, Q, S, T, Y)	54	52	53	51	52	52	51	53	51	52	51
Hydrophobic amino acid (A, I, L, F, W, V)	71	64	71	64	64	71	71	64	64	71	72
Instability index (II)	47.45	43.65	45.04	41.10	43.09	46.27	44.55	43.09	43.09	46.42	46.27
GRAVY	-0.068	0.094	-0.055	0.152	0.130	-0.078	-0.060	0.115	0.088	-0.091	-0.064
Aliphatic index	111.20	120.18	111.78	120.90	120.18	111.20	111.78	120.18	120.18	110.21	112.24

Note: Grand average of hydropathicity, GRAVY

Table S5. Predicted functional active sites within the buffalo LEP protein.

Transcripts	Amino acid positions	Sites	Details
LEP_X1	22-25	Amidation	/
	42-45	Casein kinase II phosphorylation	Phosphoserine
	77-79	Protein kinase C phosphorylation	Phosphoserine
	83-86	Casein kinase II phosphorylation	Phosphothreonine
	98-101	Casein kinase II phosphorylation	Phosphoserine
	178-181	Casein kinase II phosphorylation	Phosphoserine
LEP_X2	17-20	Casein kinase II phosphorylation	Phosphoserine
	52-54	Protein kinase C phosphorylation site	Phosphoserine
	58-61	Casein kinase II phosphorylation	Phosphothreonine
	73-76	Casein kinase II phosphorylation	Phosphoserine
	153-156	Casein kinase II phosphorylation	Phosphoserine

Table S6. Effective number of codons (ENc), GC content, and GC3s content in *LEP* gene haplotypes of buffalo.

Haplotypes	ENc	GC	GC3s
hap1_CAGG	45.24	0.587	0.758
hap2_CAGA	45.61	0.585	0.753
hap3_CGAG	45.61	0.587	0.758
hap4_CGGG	45.24	0.589	0.763
hap5_TGGG	44.42	0.587	0.763

Table S7. Relative synonymous codon usage (RSCU) values for *LEP* gene haplotypes in buffalo.

Amino acid	Codons	Hap1_CAGG	Hap2_CAGA	Hap3_CGAG	Hap4_CGGG	Hap5_TGGG
Phe	UUU	0.00	0.00	0.00	0.00	0.00
	UUC	2.00	2.00	2.00	2.00	2.00
Ser	UCU	0.32	0.32	0.32	0.32	0.32
	UCC	2.84	2.84	2.84	2.84	2.84
	UCA	0.63	0.63	0.63	0.63	0.63
	UCG	0.00	0.00	0.00	0.00	0.00
	AGU	1.26	1.26	1.26	1.26	1.26
	AGC	0.95	0.95	0.95	0.95	0.95
Tyr	UAU	0.40	0.40	0.40	0.40	0.40
	UAC	1.60	1.60	1.60	1.60	1.60
Cys	UGU	0.67	0.67	0.67	0.67	0.50
	UGC	1.33	1.33	1.33	1.33	1.50
Leu	UUA	0.19	0.19	0.19	0.19	0.19
	UUG	1.16	1.16	1.16	1.16	1.16
	CUU	0.39	0.39	0.39	0.39	0.39
	CUC	1.55	1.55	1.55	1.55	1.55
	CUA	0.19	0.39	0.39	0.19	0.19
	CUG	2.52	2.32	2.32	2.52	2.52
Pro	CCU	1.14	1.14	1.14	1.14	1.14
	CCC	2.00	2.00	2.00	2.00	2.00
	CCA	0.57	0.57	0.29	0.29	0.29
	CCG	0.29	0.29	0.57	0.57	0.57
His	CAU	0.00	0.00	0.00	0.00	0.00

Table S7 continued

Amino acid	Codons	Hap1_CAGG	Hap2_CAGA	Hap3_CGAG	Hap4_CGGG	Hap5_TGGG
His	CAC	2.00	2.00	2.00	2.00	2.00
Gln	CAA	0.43	0.43	0.43	0.43	0.43
	CAG	1.57	1.57	1.57	1.57	1.57
Arg	CGU	0.00	0.00	0.00	0.00	0.00
	CGC	0.92	0.92	0.92	0.92	0.50
	CGA	0.46	0.46	0.46	0.46	0.50
	CGG	1.38	1.38	1.38	1.38	1.50
	AGA	0.46	0.46	0.46	0.46	0.50
	AGG	2.77	2.77	2.77	2.77	3.00
Ile	AUU	0.27	0.27	0.27	0.27	0.27
	AUC	2.45	2.45	2.45	2.45	2.45
	AUA	0.27	0.27	0.27	0.27	0.27
Thr	ACU	0.44	0.44	0.44	0.44	0.44
	ACC	2.22	2.22	2.22	2.22	2.22
	ACA	0.44	0.44	0.44	0.44	0.44
	ACG	0.89	0.89	0.89	0.89	0.89
Asn	AAU	1.50	1.50	1.50	1.50	1.50
	AAC	0.50	0.50	0.50	0.50	0.50
Lys	AAA	0.75	0.75	0.75	0.75	0.75
	AAG	1.25	1.25	1.25	1.25	1.25
Val	GUU	0.00	0.00	0.00	0.00	0.00
	GUC	2.46	2.46	2.46	2.46	2.46
	GUA	0.00	0.00	0.00	0.00	0.00
	GUG	1.54	1.54	1.54	1.54	1.54
Ala	GCU	0.33	0.33	0.33	0.33	0.33
	GCC	1.67	1.67	1.67	1.67	1.67

Table S7 continued

Amino acid	Codons	Hap1_CAGG	Hap2_CAGA	Hap3_CGAG	Hap4_CGGG	Hap5_TGGG
Ala	GCA	0.67	0.67	0.67	0.67	0.67
	GCG	1.33	1.33	1.33	1.33	1.33
Asp	GAU	0.40	0.40	0.40	0.40	0.40
	GAC	1.60	1.60	1.60	1.60	1.60
Glu	GAA	0.57	0.57	0.57	0.57	0.57
	GAG	1.43	1.43	1.43	1.43	1.43
Gly	GGU	0.50	0.50	0.50	0.50	0.50
	GGC	0.50	0.50	0.50	0.50	0.50
	GGA	1.50	1.50	1.50	1.50	1.50
	GGG	1.50	1.50	1.50	1.50	1.50

Table S8. Major haplotypes identified within the buffalo *LEP* gene.

Haplotypes	Species	Actual frequency
CAGG	Swamp/River	0.3415
CAGA	Swamp/River	0.2195
CGAG	Swamp/River	0.1707
CGGG	Swamp/River	0.2561
TGGG	Swamp	0.0122

Table S9. Detailed haplotype information for the *LEP* gene in buffalo.

Haplotype	Buffalo		River buffalo		Swamp buffalo	
	Expected frequency	SE	Expected frequency	SE	Expected frequency	SE
Hap1 CAGG	0.238392	0.002621	0.058554	0.008190	0.341094	0.003406
Hap2 CAGA	0.176312	0.002580	0.102929	0.006564	0.219268	0.001715
Hap3 CAAG	0.000375	0.001904	0.003961	0.008179	0.000492	0.003161
Hap4 CAAA	0.000306	0.001956	0.001223	0.006542	0.000122	0.001217
Hap5 CGGG	0.377603	0.002689	0.588511	0.011043	0.256712	0.003371
Hap6 CGAG	0.199014	0.003135	0.244808	0.011049	0.169995	0.003565
Hap7 CGAA	0.000305	0.001736	N	N	0.000123	0.001221
Hap8 TGGG	0.007692	0.000000	N	N	0.012195	0.000000

Note: Hap: haplotype; N: none; SE: standard error.

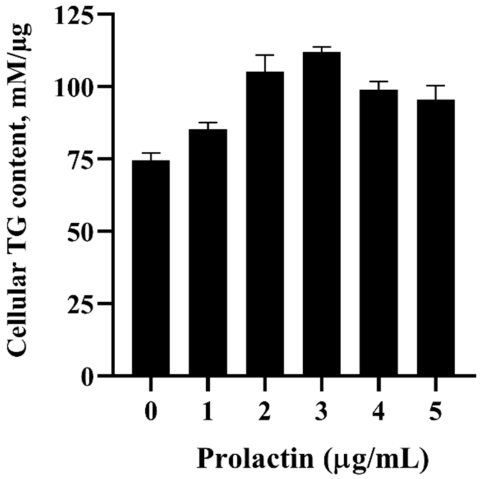

Figure S1. TG content measured across varying concentrations of prolactin.

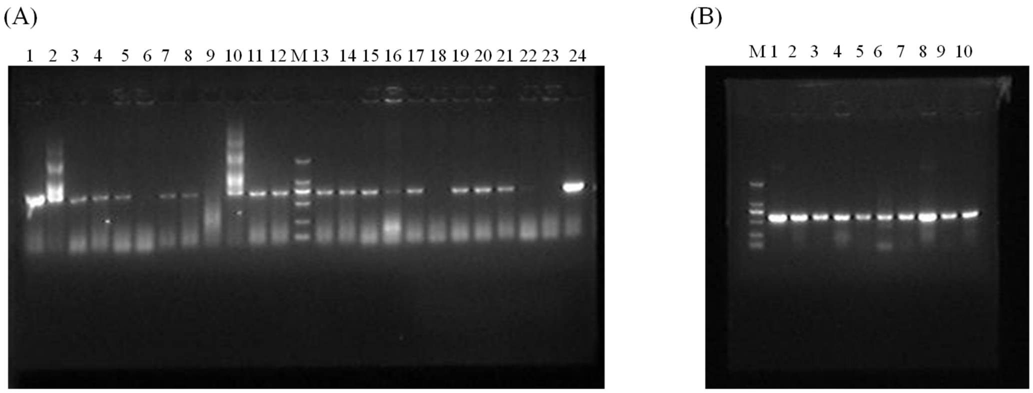

Figure S2. Original electrophoretic gels displaying the PCR results of buffalo *LEP*_X1_pMD18-T (A) and *LEP*_X2_pMD18-T (B) clones, with respective lengths of 704 bp and 600 bp. M, Marker-DL2000; lanes 1-24: clones of *LEP*_X1_pMD18-T (A); lanes 1-10: clones of *LEP*_X2_pMD18-T (B). The skewed lines observed in the electrophoretic gels are attributed to misaligned electrodes within the electrophoresis bath, while the smearing lines are indicative of false-positive clones.

Percent identity

Divergence

	1	2	3	4	5	6	7	8	9	10	11	12	13	14	15	16	17	18	19		
1	***	99.4	99.4	98.2	98.8	98.8	97.6	98.8	98.2	97.6	97.6	83.2	83.8	84.4	92.2	93.4	93.4	93.4	98.2	1	*Bubalus_bubalis_* X2
2	0.6	***	100	98.8	99.5	99.5	98.2	99.4	98.8	97.9	98.4	82.6	83.2	84.4	86.8	92.7	88.7	92.2	98.8	2	*Bubalus_bubalis_* X1
3	0.6	0	***	98.8	99.5	99.5	98.2	99.4	98.8	97.9	98.4	82.5	83.1	84.3	86.7	92.7	88.6	92.1	98.8	3	*Bubalus_bubalis_* X3
4	1.8	1.2	1.2	***	99.4	99.4	98.8	99.4	99.4	98.2	98.2	83.2	83.8	84.4	90.4	93.4	93.4	93.4	100	4	*Bos_taurus_* X2
5	1.2	0.5	0.5	0.6	***	100	98.8	100	99.4	98.4	99	83.2	83.8	84.4	86.2	93.2	89.2	92.7	99.4	5	*Bos_indicus_* X1
6	1.2	0.5	0.5	0.6	0	***	98.8	100	99.4	98.4	99	83.1	83.7	84.3	86.2	93.2	89.2	92.7	99.4	6	*Bos_indicus_* X2
7	2.4	1.8	1.8	1.2	1.2	1.2	***	98.8	99.4	97.6	97.6	82.6	83.2	84.4	89.8	92.8	92.8	92.8	98.8	7	*Bos_mutus*
8	1.2	0.6	0.6	0.6	0	0	1.2	***	99.4	98.8	98.8	83.2	83.8	84.4	91	93.4	93.4	93.4	99.4	8	*Bison_bison*
9	1.8	1.2	1.2	0.6	0.6	0.6	0.6	0.6	***	98.2	98.2	83.2	83.8	84.4	90.4	93.4	93.4	93.4	99.4	9	*Bos_frontalis*
10	2.4	2.1	2.1	1.8	1.6	1.6	2.4	1.2	1.8	***	99.5	82.6	83.2	84.4	86.2	92.7	88.7	92.2	98.2	10	*Ovis_aries*
11	2.4	1.6	1.6	1.8	1	1.1	2.4	1.2	1.8	0.5	***	82.6	83.2	84.4	86.2	93.2	89.2	92.7	98.2	11	*Capra_hircus*
12	19	19.8	19.9	19	19	19.2	19.8	19	19	19.8	19.8	***	96.4	83.2	82.6	83.8	83.8	83.8	83.1	12	*Mus_musculus*
13	18.3	19	19.2	18.3	18.3	18.4	19	18.3	18.3	19	19	3.7	***	82	83.2	83.8	83.8	83.8	83.7	13	*Rattus_norvegicus*
14	17.5	17.5	17.6	17.5	17.5	17.6	17.5	17.5	17.5	17.5	17.5	19	20.6	***	88	86.2	86.2	86.2	84.3	14	*Homo_sapiens*
15	8.2	14.6	14.7	10.3	15.2	15.3	11	9.6	10.3	15.2	15.2	19.8	19	13.1	***	88.9	88	88.9	90.4	15	*Equus_asinus*
16	6.9	7.7	7.7	6.9	7.1	7.1	7.6	6.9	6.9	7.7	7.1	18.3	18.3	15.3	12.1	***	95.7	99.5	93.4	16	*Camelus_bactrianus*
17	6.9	12.3	12.3	6.9	11.6	11.7	7.6	6.9	6.9	12.3	11.6	18.3	18.3	15.3	13.1	4.4	***	95.7	93.4	17	*Camelus_dromedarius*
18	6.9	8.3	8.3	6.9	7.7	7.7	7.6	6.9	6.9	8.3	7.7	18.3	18.3	15.3	12.1	0.5	4.4	***	93.4	18	*Vicugna_pacos*
19	1.8	1.2	1.2	0	0.6	0.6	1.2	0.6	0.6	1.8	1.8	19.2	18.4	17.6	10.3	7	7	7	***	19	*Bos_taurus* _X1
	1	2	3	4	5	6	7	8	9	10	11	12	13	14	15	16	17	18	19		

Figure S3. Percent identity/divergence amino acid sequences of LEP between Bovidae and non-Bovidae species.

```
(A)          10        20        30        40        50        60        70
              |         |         |         |         |         |         |
MEPRRDRGIAAPAAARPQKPIPGRKMRCGPLYQFLWLWPYLSYVEAVPIRKVQDDTKTLIKTIVTRINDI
ccccccttccccccccccccccccccccccccheeeeecccccccccchhhcchhhhhhhheeeehhccc
SHTQSVSSKQRVTGLDFIPGLHPLLSLSKMDQTLAIYQQILTSLPSRNVVQISNDLENLRDLLHLLAASK
cccccccccccettceecccccchhhhhhhhhhhhhhhhhhhhccccchhhhhhhhhhhhhhhhhhhhht
SCPLPQVRALESLESLGVVLEASLYSTEVVALSRLQGSLQDMLQQLDLSPGC
tcccccccchhhhhhhhhhhhhhhhhhhhhhhhhhhhhhhhhhhhhtccttc
```

```
(B)          10        20        30        40        50        60        70
              |         |         |         |         |         |         |
MRCGPLYQFLWLWPYLSYVEAVPIRKVQDDTKTLIKTIVTRINDISHTQSVSSKQRVTGLDFIPGLHPLL
cttcchhhheehhcccccccccchhccctthhhhhhheeeehccccccccccccccettceecccccchh
SLSKMDQTLAIYQQILTSLPSRNVVQISNDLENLRDLLHLLAASKSCPLPQVRALESLESLGVVLEASLY
hhhhhhhhhhhhhhhhhhccccchhhhhhhhhhhhhhhhhhhhhtcccccccchhhhhhhhhhhhhhhhh
STEVVALSRLQGSLQDMLQQLDLSPGC
hhhhhhhhhhhhhhhhhhhhhtccttc
```

```
(C)          10        20        30        40        50        60        70
              |         |         |         |         |         |         |
MRCGPLYQFLWLWPYLSYVEAVPIRKVQDDTKTLIKTIVTRINDISHTQSVSSKQRVTGLDFIPGLHPLL
cttcchhhheehhcccccccccchhccctthhhhhhheeeehccccccccccccccettceecccccchh
SLSMDQTLAIYQQILTSLPSRNVVQISNDLENLRDLLHLLAASKSCPLPQVRALESLESLGVVLEASLYS
hhhhhhhhhhhhhhhhhccccchhhhhhhhhhhhhhhhhhhhhtcccccccchhhhhhhhhhhhhhhhhh
TEVVALSRLQGSLQDMLQQLDLSPGC
hhhhhhhhhhhhhhhhhhhhtccttc
```

Figure S4. Predicted secondary structure of buffalo LEP_X1 (A), LEP_X2 (B) and LEP_X3 (C). Alpha helices, β turn, extended strands and random coils are indicated with h, t, e and c, respectively.

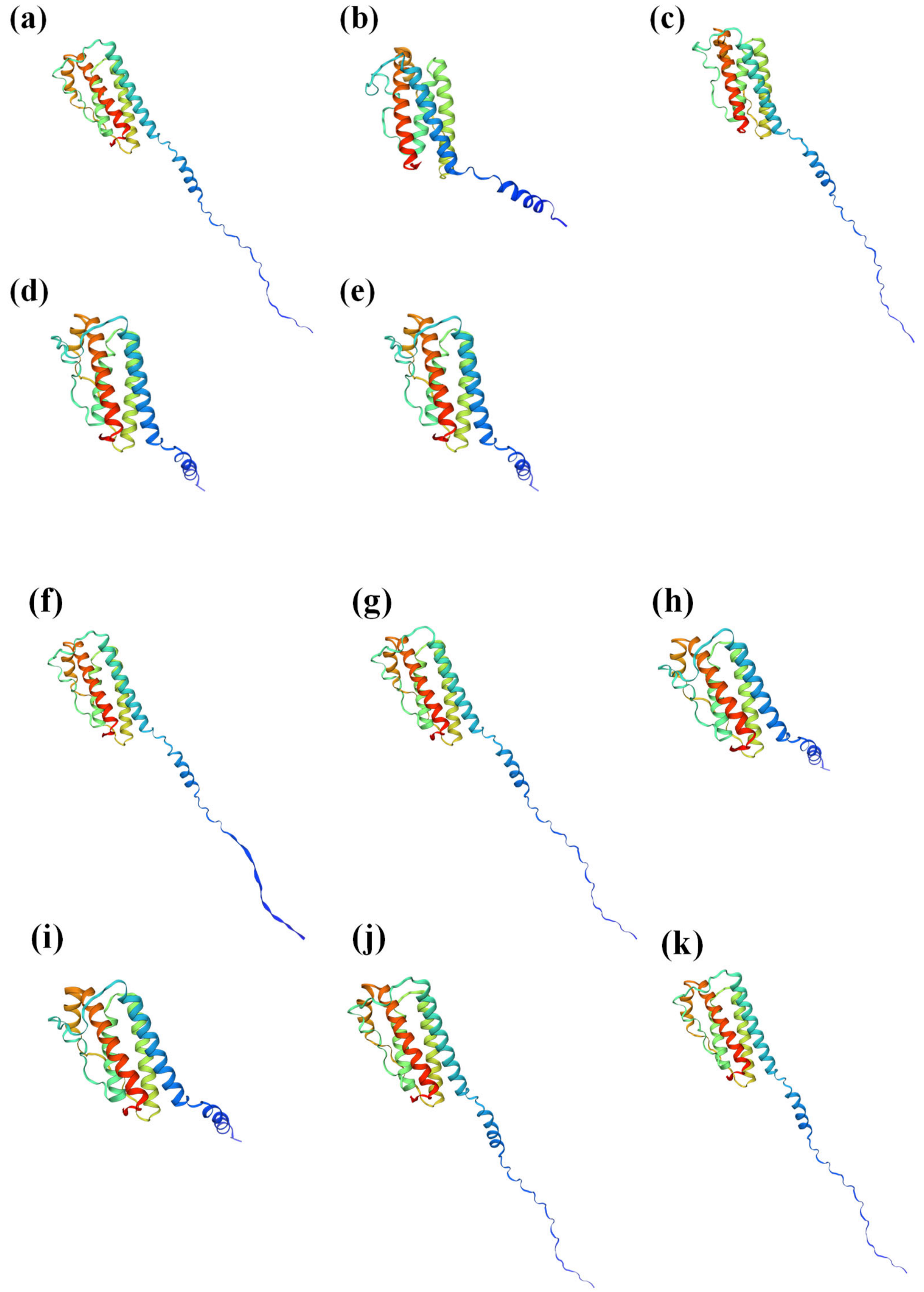

Figure S5. Comparative analysis of the tertiary structures of LEP variants across different species: LEP_X1, LEP_X2, LEP_X3 in buffalo (a-c), LEP_X1, LEP_X2 in cattle (d-e), LEP_X1, LEP_X2 in zebu (f-g), LEP_X2 in bison (h) and yak (i), and LEP_X1 in goat (j) and sheep (k).

				111	111	111	122	222	222	223	333	333	333	444	444	444	455	555	555	556	666	666	666	777	777	889
	123	456	789	012	345	678	901	234	567	890	123	456	789	012	345	678	901	234	567	890	123	456	789	012	345	890
**hap1_CAGG**	ATG	GAA	CCC	CGG	AGG	GAT	CGA	GGA	ATC	GCG	GCG	CCA	GCA	GCA	GCG	AGG	CCC	CAG	AAG	CCC	ATC	CCG	GGA	AGG	AAA	CCC
**hap2_CAGA**	...	...	...	...	...	...	...	...	...	...	...	...	...	...	...	...	...	...	...	...	...	...	...	...	...	...
**hap3_CGAG**	...	...	...	...	...	...	...	...	...	...	...	...	...	...	...	...	...	...	...	...	...	...	...	...	...	...
**hap4_CGGG**	...	...	...	...	...	...	...	...	...	...	...	...	...	...	...	...	...	...	...	...	...	...	...	...	...	...
**hap5_TGGG**	...	...	...	...	...	...	...	...	...	...	...	...	...	...	...	...	...	...	...	...	...	...	...	...	...	...
***Bubalus_bubalis* _XM_044946369.2**	...	...	...	...	...	...	...	...	...	...	...	...	...	...	...	...	...	...	...	...	...	...	...	...	...	...
***Bubalus_bubalis* _XM_044946370.2**	...	...	...	...	...	...	...	...	...	...	...	...	...	...	...	...	...	...	...	...	...	...	...	...	...	...
***Bos_taurus* _XM_010804455.4**	---	---	---	---	---	---	---	---	---	---	---	---	---	---	---	---	---	---	---	---	---	---	---	---	---	...
***Bos_taurus* _NM_173928.2**	---	---	---	---	---	---	---	---	---	---	---	---	---	---	---	---	---	---	---	---	---	---	---	---	---	...
***Bos_indicus* _XM_019959033.1**	...	..G	...	...	...	...	...	...	...	...	...	...	...	..G	...	...	...	...	...	...	...	...	...	...	...	...
***Bos_indicus* _XM_019959034.1**	...	..G	...	...	...	...	...	...	...	...	...	...	...	..G	...	...	...	...	...	...	...	...	...	...	...	...
***Bison_bison_bison*_ XM_010831717.1**	---	---	---	---	---	---	---	---	---	---	---	---	---	---	---	---	---	---	---	---	---	---	---	---	---	...
***Bos_mutus* _XM_005893666.1**	---	---	---	---	---	---	---	---	---	---	---	---	---	---	---	---	---	---	---	---	---	---	---	---	---	T..
***Capra_hircus* _XM_018046968.1**	...	..G	...	...	...	...	...	...	...	...	...	...	...	..G	...	...	...	...	...	...	...	...	...	...	...	...
***Ovis_aries* _XM_027968780.2**	...	..G	...	...	...	...	...	...	...	...	...	...	...	..G	...	...	...	...	...	...	.C.	...	...	...	...	...

			111	111	111	111	222	333	333	333	333	333	444	444	444	444	444	444	444	455	555	555	555	555	555
	999	999	222	222	445	888	222	111	111	333	556	666	333	333	445	555	666	777	888	900	222	333	555	666	667
	456	789	123	456	890	456	012	012	345	456	890	789	012	345	890	456	678	234	456	901	345	234	012	567	890
**hap1_CAGG**	TAC	CAA	CTG	TCC	CGC	ACG	CAG	TTA	GCG	ACC	GTC	TCT	TTG	CCA	CTG	AGT	TTG	GTC	GCC	ACC	CTG	TCA	CAG	AGT	CCT
**hap2_CAGA**	...	...	...	...	...	...	...	...	...	...	...	...	...	...	...	...	...	...	...	...	...	...	...	...	...
**hap3_CGAG**	...	...	...	...	...	...	...	...	...	...	...	...	...	..G	..A	...	...	...	...	...	...	...	...	...	...
**hap4_CGGG**	...	...	...	...	...	...	...	...	...	...	...	...	...	..G	...	...	...	...	...	...	...	...	...	...	...
**hap5_TGGG**	...	...	...	...	T..	...	...	...	...	...	...	...	...	..G	...	...	...	...	...	...	...	...	...	...	...
***Bubalus_bubalis* _XM_044946369.2**	...	...	...	...	...	...	...	..G	...	...	...	...	...	...	...	...	...	...	...	...	...	...	.G.	...	...
***Bubalus_bubalis* _XM_044946370.2**	...	...	...	...	...	...	---	..G	...	...	...	...	...	...	...	...	...	...	...	...	...	...	.G.	...	...
***Bos_taurus* _XM_010804455.4**	..T	.G.	...	..T	T..	..A	---	..G	...	...	...	..C	...	..G	...	..C	...	..T	..T	...	...	...	.G.	...	..C
***Bos_taurus* _NM_173928.2**	..T	.G.	...	..T	T..	..A	...	..G	...	...	...	..C	...	..G	...	..C	...	..T	..T	...	...	...	.G.	...	..C
***Bos_indicus* _XM_019959033.1**	..T	.G.	...	..T	...	..A	...	..G	...	...	...	..C	...	..G	...	..C	...	...	..Y	...	...	...	.G.	...	...
***Bos_indicus* _XM_019959034.1**	..T	.G.	...	..T	...	..A	---	..G	...	...	...	..C	...	..G	...	..C	...	...	..Y	...	...	...	.G.	...	...
***Bison_bison_bison*_ XM_010831717.1**	..T	.G.	...	..T	...	..A	...	..G	...	...	...	..C	...	..G	...	..C	...	...	..T	..T	...	...	.G.	...	...
***Bos_mutus* _XM_005893666.1**	..T	.G.	...	...	A..	...	...	..G	...	...	...	..C	...	..G	...	..C	...	...	..T	..T	...	...	.G.	...	...
***Capra_hircus* _XM_018046968.1**	...	.G.	..C	...	...	...	...	..G	..A	G..	A..	...	..A	...	...	..C	C..	...	...	...	..A	..T	.G.	..C	...
***Ovis_aries* _XM_027968780.2**	...	.G.	..C	...	...	...	...	..G	..A	G..	A..	...	...	..G	...	..C	C..	...	...	...	..A	..T	.G.	..C	...

Note: Numbers represent positions. Different letters represent different nucleotides. Dots (·) indicate identity with hap1_CAGG. Horizontal bars (-) mean nucleotides deletion.

Figure S6. Nucleotide sequence differences in LEP among Bovidae species.

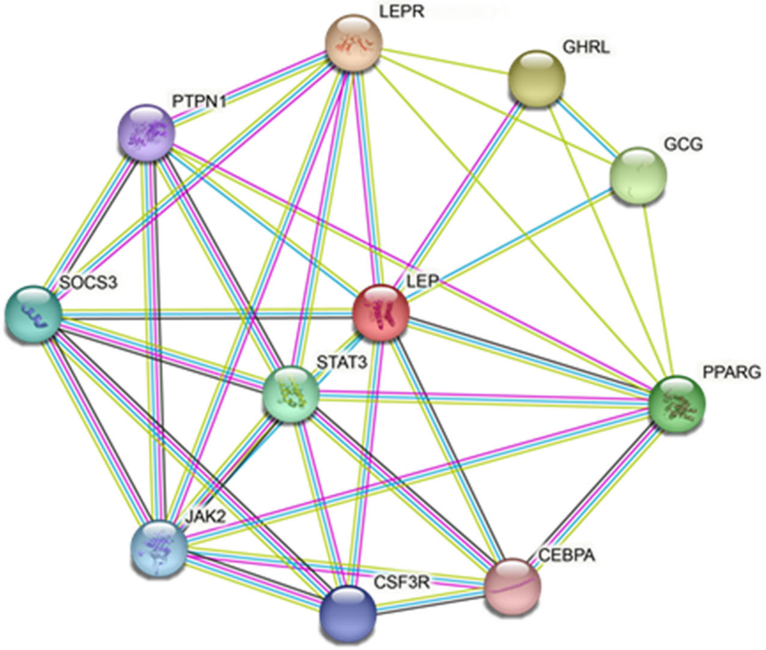

Figure S7. Protein interactions network involving LEP in buffalo.
